# Comprehensive proteomic characterization of urethral stricture disease in the Chinese population

**DOI:** 10.3389/fmolb.2024.1401970

**Published:** 2024-07-26

**Authors:** Jiangtao Gao, Hui Liu, Lingling Li, Chunmei Guo, Zhiyong Wang, Mengya Cheng, Subei Tan, Lu Chen, Jijing Shi, Hui Wu, Chao Feng, Guoying Yu, Chen Ding

**Affiliations:** ^1^ Department of Urology, The First People’s Hospital of Zhengzhou, Henan, China; ^2^ State Key Laboratory of Genetic Engineering and Collaborative Innovation Center for Genetics and Development, School of Life Sciences, Human Phenome Institute, Fudan University, Shanghai, China; ^3^ State Key Laboratory Cell Differentiation and Regulation, Overseas Expertise Introduction Center for Discipline Innovation of Pulmonary Fibrosis, (111 Project), College of Life Science, Henan Normal University, Xinxiang, China; ^4^ Key Medical Laboratory of Stem Cell Transformation and Application, Department of Pathology, The First People’s Hospital of Zhengzhou, Henan, China; ^5^ Department of Urology, Shanghai Jiao Tong University Affiliated Sixth People’s Hospital, Shanghai, China; ^6^ Institute of Cancer Research, Affiliated Tumor Hospital of Xinjiang Medical University, Urumqi, China

**Keywords:** proteomics, USD, scar formation, immune infiltration, biomarkers

## Abstract

**Background:**

Male urethral stricture disease (USD) is predominantly characterized by scar formation. There are few effective therapeutic drugs, and comprehensive molecular characterizations of USD formation remain undefined.

**Methods:**

The proteomic profiling of twelve scar tissues and five matched normal adjacent tissues (NATs). Proteomic analysis methods were applied to explore the molecular characterizations of USD formation, including uncovering mechanistic pathways and providing novel biomarkers for scar formation.

**Results:**

Comparative proteomic analysis showed that the extracellular matrix (ECM) and complement cascade signaling were predominant in scar tissues. COL11A1 and CD248 significantly contributed to the accumulation of ECM components. Our study presented diverse molecular mechanisms of scar formation across different ages and suggested the potential effects of PXK in Age 1 (<45) patients. Furthermore, immune infiltration studies indicated the therapeutic potential of inhibiting the complement system (C4A, C4B) in Age 2 (≥45) patients, providing a potential clinical strategy for USD.

**Conclusion:**

This study illustrated the pathogenesis of USD formation and the diverse characteristics of USD patients with different ages, enhancing our understanding of the disease’s pathogenesis and providing a valuable resource for USD treatment.

## Introduction

Urethral stricture disease (USD) is common following urethral injury associated with scar formation and fibrosis in sub-epithelial tissue (Zhang et al., 2018; Lumen et al., 2021); it affects approximately 300 per 100,000 men (Santucci et al., 2007; Gelman and Furr, 2020). The etiology of USD is diverse, with iatrogenic causes predominant in the Western world (Stein et al., 2013) while infectious strictures due to venereal infections or a non-specific urethritis are more common in developing countries (Fall et al., 2011). USD significantly impacts patients’ quality of life and healthcare costs (Bertrand et al., 2015). In addition, USD can lead to lower urinary tract symptoms and life-threatening complications, such as hydronephrosis (∼20% incidence) and acute retention (60% incidence) (Hampson et al., 2014). Generally, open urethroplasty has become the gold standard for treating urethral strictures but requires expert surgeons and high-volume urethral referral centers (Hampson et al., 2014). Notwithstanding, a high recurrence rate (∼60%) is still a challenge for USD (Lumen et al., 2011), and thus novel medicative biomarkers are urgently needed.

Trauma or urological surgery (invasive procedures) is associated with urethral injury that initiates an inflammatory response followed by tissue repair, including fibrotic remodeling (Wan et al., 2018; Cheng et al., 2021). The fibrotic process involves the overgrowth, hardening, and/or scarring of various tissues, and it is characterized by the excessive deposition of extracellular matrix (ECM) proteins such as collagen, fibronectin, and hyaluronic acid in surrounding tissues (Wynn, 2008; Daud et al., 2021; Kumari et al., 2022). There is a hypothesis that the scar formation and subsequent contraction after surgery are the primary reason for USD and its recurrence (Perdzynski and Adamek, 2020), implying an effective potential treatment for USD prevention. However, the molecule characterization of scar formation in USD is currently unknown.

Proteomics is a crucial tool for unraveling the molecular mechanism behind biological systems and is utilized for basic and applied research. The high recurrence rate of USD indicates the lack of effective drugs, which negatively impacts patients’ quality of life. Thus, the precise pathogenesis of USD is responsible for potential therapeutic targets and clinical diagnostic markers. However, the molecular characterizations of scar formation and tissue remodeling are undefined at the protein level. Notably, the incidence of urethral stricture scarring in males increases significantly after the age of 55, with a mean onset age of 45.1 (Palminteri et al., 2013; Alwaal et al., 2014), underscoring age as a critical risk factor for USD. Immune response is pivotal in the pathogenesis of urethral strictures. Following tissue injury, it initiates an inflammatory response that is essential for tissue repair. However, an excessive or prolonged inflammatory response can lead to aberrant scar formation and fibrosis (Barnes et al., 2015), which are characteristics of urethral strictures. In this study, we performed proteomic profiling of USD, elucidating the molecular mechanism of scar formation. In addition, we disclosed the scar molecule features of USD patients with different ages and illustrated the immune portraits of scar formation, thus providing a reference database and potential biomarkers for USD.

## Materials and methods

### Patient samples of USD

Urethral scar tissues and matched normal adjacent tissues were prospectively collected from July to August 2021 at the Sixth People’s Hospital Affiliated to Shanghai Jiao Tong University. There were no biases in selecting the patients, and none of them had received any prior treatment for urethral scarring prior to surgical excision. All samples were individually dissected from formalin-fixed, paraffin-embedded (FFPE) slides and assessed by two or three experienced urological pathologists, who marked the hematoxylin and eosin (H&E)-stained sections (3 μm-thick) of the FFPE slides. Clinical information of the USD patients, including age, gender, and history of present illness (HPI), is listed in Supplementary Table S1.

### Laser-capture microdissection

In our cohort, we applied laser-capture microdissection (LCM; Emmert-Buck et al., 1996) to precisely dissect the sections of NAT/Scar samples. All FFPE specimens were deparaffinized with xylene and then rehydrated through graded alcohol and water. The H&E sections were stained with Mayer’s hematoxylin (Sigma) and dehydrated through graded alcohol and xylene. Before microdissection, FFPE specimens were sectioned with a microtome (10 μm thick) and mounted from FFPE blocks, and they were then micro-dissected with a Leica LMD 6500 laser microdissection system, depending on the FFPE specimen evaluated by two or three experienced pathologists in advance. The NAT/Scar samples were collected in 1.5-mL tubes and kept in storage at −80 °C until further processing. These methods are also applied in other published proteogenomic studies, such as in duodenal cancer (Lingling et al., 2023a) and glioblastoma (Lam et al., 2022).

### Protein extraction and digestion

Nearly 50 μL TCEP buffer (2% deoxycholic acid sodium salt, 40 mM 2-chloroacetamide, 100 mM tris-phosphine hydrochloride, 10 mM (2-carboxyl)-phosphine hydrochloride, 1 mM phenylmethylsulfonyl fluoride mixed with MS water, pH 8.5) was added to 1.5 mL EP tubes (Eppendorf tubes) with prepared samples (0.01 cm × 0.01 cm) and then heated in a 99 °C metal bath for 30 min. After cooling to room temperature, a BCA protein concentration determination kit (Thermo) was used to determine the protein concentration of each sample, and the concentration was adjusted to be uniform across all samples. Taking the same amount of protein from the NATs and scar tissues for digestion, 3 μg trypsin (REF: V528A, PROMEGA) was added to each tube and digested for 18 h in a 37 °C incubator. Subsequently, 13 μL 10% formic acid was added to each tube and vortexed for 3 min, followed by sedimentation for 5 min at 12,000 g. After that, a new 1.5 mL tube with 350 μL buffer (0.1% formic acid in 50% acetonitrile) was used to collect the supernatant for extraction (vortex for 3 min, and then 12,000 g sedimentation for 5 min). The supernatant was then transferred into a new tube for drying in a 60 °C vacuum drier. After drying, 100 μL 0.1% formic acid was needed to dissolve the peptides, with vortexing for 3 min and then sedimentation for 3 min at 12,000 g. The supernatant was transferred to a new tube and then desalinated. Before desalination, the activation of columns with tow slides of 3M C18 disk was required, with the lipid as follows: 90 μL 100% acetonitrile twice, 90 μL 50% and 80% acetonitrile once in turn, and then 90 μL 50% acetonitrile once. After pillar balance with 90 μL 0.1% formic acid twice, the supernatant of the tubes was loaded into the pillar twice and decontaminated with 90 μL 0.1% formic acid twice. Finally, 90 μL elution buffer (0.1% formic acid in 50% acetonitrile) was added to the pillar for elution twice. The collected liquid was then dried in a 60 °C vacuum drier. The nominal mass of peptides in the sample was estimated based on BCA quantification of the lysate prior to digestion, and 2 µg peptides of every sample were injected for LC-MS/MS analysis.

### Liquid chromatography (LC)-mass spectrometry (MS)/MS Analysis

For proteomic sample profiling, peptides were analyzed on a Q Exactive HF-X Hybrid Quadrupole-Orbitrap Mass Spectrometer (Thermo Fisher Scientific, Rockford, IL, United states) coupled with a high-performance liquid chromatography system (EASY nLC 1200, Thermo Fisher). Dried peptide samples re-dissolved in Solvent A (0.1% FA in water) were loaded onto a 2-cm self-packed trap column (100-μm inner diameter, 3 μm ReproSil-Pur C18-AQ beads, Dr. Maisch GmbH) and separated on a 150-μm-inner-diameter column 30 cm long (1.9 μm ReproSil-Pur C18-AQ beads, Dr. Maisch GmbH) over 150 min. The eluted peptides were ionized at 2.0 kV and introduced into the mass spectrometer. MS was performed in a data-dependent acquisition mode. For the MS1 spectra full scan, ions with 300–1,400 m/z were acquired by an Orbitrap mass analyzer at a 120,000high resolution. The automatic gain control (AGC) target value was set as 3E6 and the maximal ion injection time was 80 ms. MS2 Spectra acquisition was conducted using the Orbitrap analyzer, with precursor ions selected and fragmented by higher-energy collision dissociation (HCD) at a normalized collision energy of 27%. Fragment ions were analyzed using the Orbitrap with the AGC target set at 5E4. The maximal ion injection time of MS2 was 20 ms. Peptides that triggered MS/MS scans were dynamically excluded from further MS/MS scans for 12 s.

### Quality control of the MS platform

For quality control (QC) of MS performance, tryptic digests of HEK293T cell lysates were measured as a QC standard every 2 days. The QC samples were run using the same method with USD samples, including conditions, software, and MS parameters. Pairwise Spearman’s correlation coefficients were calculated using the R package corrplot (v 0.84) for all QC runs (Supplementary Figure S1B). The average correlation coefficient among standards was 0.96, with a maximum of 0.99 and minimum of 0.92, indicating that MS was robust and consistent.

### Proteome identification and quantification

The proteome raw datasets generalized in this study have been deposited with the ProteomeXchange Consortium (dataset identifier: PXD053186) via the iProX partner repository (https://www.iprox.cn/) (Jie et al., 2018; Tao et al., 2021) under Project ID IPX0003849000.

Raw files were processed on the Firmiana platform (a one-stop proteomic cloud platform, https://phenomics.fudan.edu.cn/firmiana/) (Feng et al., 2017) and searched against the human National Center for Biotechnology Information (NCBI) RefSeq protein database (updated on 04-07-2013, 32,015 entries) using the Mascot 2.4 search engine (Matrix Science Inc). The statistical significance of differences between the two groups was calculated with the Wilcoxon rank-sum test. Pearson’s correlation coefficient was used for the correlation analysis between the two proteins. Statistical significance was considered when *p* < 0.05.

The maximum number of missed cleavages was set to 2. A mass tolerance of 20 ppm for precursor and 0.05 Da for production was allowed. Carbamidomethyl (C) was considered as a fixed modification. For the proteome profiling data, variable modifications were oxidation (M) and acetylation (Protein N-term). For the QC of protein identification, the target-decoy based strategy was applied to ensure that the FDR (false discovery rate) of both peptide and protein remained lower than 1%. A percolator was used to obtain the probability value (q-value), validating that the FDR (measured by decoy hits) of every peptide–spectrum match (PSM) remained lower than 1%. All peptides shorter than seven amino acids were then removed. The cutoff ion score for peptide identification was 20. All PSMs in all fractions were combined for protein quality control—a stringent quality control strategy. The q-values of both target and decoy peptide sequences were dynamically increased until the corresponding protein FDR was less than 1% employing the parsimony principle. Finally, to reduce the false positive rate, the proteins with at least one unique peptide were selected for further investigation

Label-free protein quantifications were calculated using a label-free, intensity-based absolute quantification (iBAQ) approach (Wei et al., 2012). The FOT represents the normalized abundance of a particular protein across samples, calculated by a protein’s iBAQ divided by the total iBAQ of all proteins identified in each sample. To facilitate presentation, FOT values were multiplied by 10^5^, and missing values were imputed as half the minimum values (Katharine Nichole et al., 2021; Qin et al., 2022). This quantification approach was applied in our previous studies in duodenal (Lingling et al., 2023a) and esophageal (Lingling et al., 2023b) cancers.

### Missing values imputation

For the missing values (NAs), we firstly applied the match-between-runs (MBR) algorithm (Wan et al., 2018). We built a dynamic regression function based on commonly identified peptides in samples. According to correlation value R^2^, the function chooses a linear or quadratic function for regression to calculate the retention time (RT) of corresponding hidden peptides and checks the existence of the extracted ion chromatogram (XIC) based on the m/z and calculated RT. The function evaluated the peak area values of those existing XICs. These peak area values are considered parts of corresponding proteins. MBR has been proved to be an effective technique to fill the missing values and has widely been used in other proteomic studies (Wynn, 2008). As for the rest of the missing values after applying MBR, to avoid artificially increasing the FDR we did not apply other data imputation algorithms. Missing values were assigned to half of the minimum value across our proteome data. This strategy has been applied in previous studies, such as the fragile X-associated tremor/ataxia syndrome project (Bertrand et al., 2015) and in thyrotoxicosis mice (Hampson et al., 2014).

### Principal component analysis of trace FFPE samples

We performed principal component analysis (PCA) on the identified proteins to illustrate the global proteomic difference between NATs and scar tissues. The PCA function under the scikit-learn R package was implemented for unsupervised clustering analysis with the parameter “n_components = 2” on the expression matrix of global proteomic data.

### Differential proteomics analysis

#### Scar-associated proteomic events

A total of 17 samples from 12 USD patients for proteomic profiling were used, and the proteome data were processed as follows. All proteins were required to have at least two unique strict peptides, and missing values were imputed as the half of the minimum value across our proteome data. A total of 5,741 protein groups were identified with a 1% FDR at both protein and peptide levels. Comparing the features of the NATs and scar tissues, the DEPs of the NATs and scar tissues were identified using SAM analysis. The DEPs were defined by following criteria: *p*-value less than 0.05 (Wilcoxon rank-sum test) (Zhang et al., 2018), and fold change (scar tissues vs. NATs ratio) was either no less than 2 (≥2) or no more than 0.5 (≤0.5) (Lumen et al., 2021). Consequently, 155 elevated (scar proteins) and 417 descended proteins (NATs proteins) were detected. The makers of fibroblasts and myofibroblasts (referenced in the CellMarker web: http://bio-bigdata.hrbmu.edu.cn/CellMarker/) investigate how urethral injury causes fibrotic scars and the potential therapeutic targets. We integrated the highly expressed proteins (n = 1,145, fold change (scar tissues/NATs) > 2)) of the scar tissues which were then matched to the makers of fibroblasts and myofibroblasts. The same methods are also applied in other proteogenomic studies, such as in duodenal (Lingling et al., 2023a) and esophageal (Lingling et al., 2023b) cancer.

#### Proteomic profiles associated with ages of USD patients

For differential analysis of proteins in Age 1 and 2 patients, the highly expressed proteins of each age were screened. For paired samples of NATs and scar tissue, we used the ratio of scar to NAT expression levels from the same patient. For unpaired samples, we calculated the expression level of scar relative to the average NAT expression across all other patients. This method, successfully applied in our previous research on cholangiocarcinoma (Mengjie et al., 2022), ensured that our analysis accommodated the clinical reality of sample collection. The Age 1 proteins were defined thus: fold change (scar tissues vs. NATs ratio) greater than 2 (Zhang et al., 2018) and fold change (Age 1 vs. Age 2 ratio) no less than 2 (≥2) (Lumen et al., 2021). The Age 2 proteins were defined by the following criteria: fold change (scar tissues vs. NATs ratio) greater than 2(Zhang et al., 2018) and fold change (Age 1 vs. Age 2 ratio) no more than 0.5 (≤0.5) (Lumen et al., 2021). We integrated the Age 1 specific proteins (n = 477) and the actin binding proteins (n = 266) (referenced in the AmiGO 2 web: http://amigo.geneontology.org/) to investigate the potential markers of the Age 1 patients. The Age 1 potential markers were defined by following criteria: proteins were identified both in Age 1 specific proteins and the actin binding proteins (Zhang et al., 2018), *p*-value less than 0.05 (Wilcoxon rank-sum test) (Lumen et al., 2021), fold change (scar tissues vs. NATs ratio) greater than 2 (Gelman and Furr, 2020), and fold change (Age 1 vs. Age 2 ratio) no less than 2 (≥2) (Santucci et al., 2007).

#### Immune characterizations of USD

To investigate immune infiltration in USD, we utilized the Human Protein Atlas (HPA) immune protein database (https://www.proteinatlas.org). Specific immune-related proteins, such as interleukins and interferons, were searched by entering their names into the website’s search function. Differential analysis was performed with the Wilcoxon rank-sum test to determine the differential abundance of proteins between two groups (e.g., scar tissue vs. NATs; Age 1 vs. Age 2). Proteins with no NAs in at least 20% of samples were considered in each group. We consequently identified 31 and 17 significant immune proteins in NATs/scar tissues and Age 1/Age 2 patients, respectively.

### Biological pathways enrichment analysis

To investigate the dominant signaling pathways of the DEPs between the NATs and scar tissues and between Age 1 (<45) and Age 2 (≥45) USD patients, we utilized gens sets of molecular pathways in DAVID (Kanehisa and Goto, 2000). Pathways from the GOBP/KEGG database were considered for this analysis. Statistical significance was assumed when the *p*-value was less than 0.05.

### Immunohistochemistry analysis

To measure the expression of COL11A1 and PXK protein in tissue by immunohistochemistry (IHC) staining, 3-μm-thick sections from each formalin-fixed, paraffin-embedded (FFPE) tissue block were de-waxed with xylene and rehydrated through a graded series of ethanols, as prepared by the Sixth People’s Hospital Affiliated to Shanghai Jiao Tong University. Heat-induced antigen retrieval steps were performed at pH 9.0 for all targets. Antibodies CD248 (1:2000, Abcam, catalog No: Ab204914) and PXK (1:200, Bioss antibodies, catalog No: bs-19688R) were incubated at room temperature for 1 h followed by standard chromogenic staining.

## Results

### Proteomic landscape of USD

**FIGURE 1:**
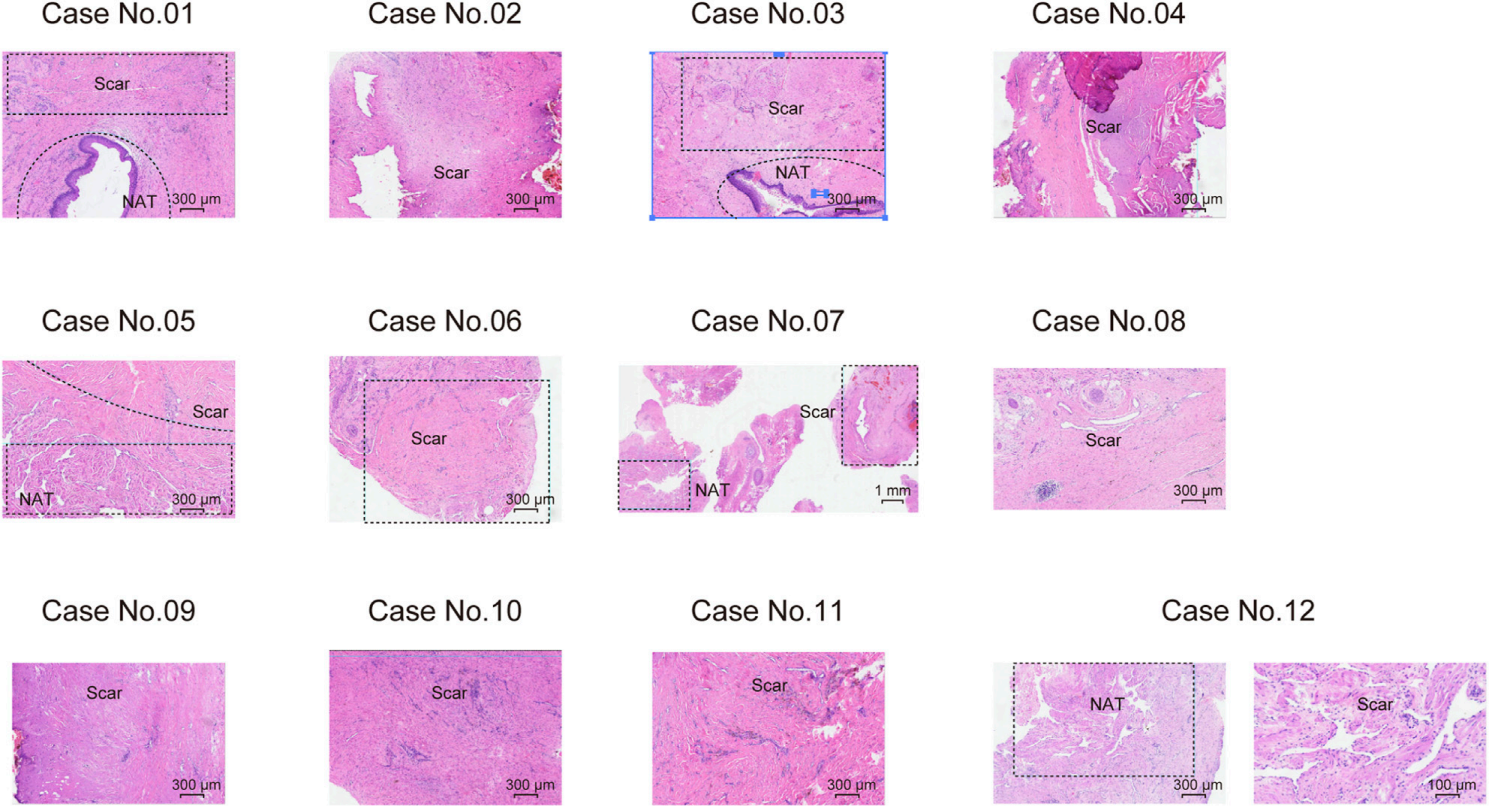
Haematoxylin and eosin (H&E) staining showing the morphological characteristics of scar tissues and NATs on 12 male USD patients.

We prospectively collected samples from twelve treatment-naive male USD patients, and five scar tissues matched normal adjacent tissues (NATs). Precise LCM (Emmert-Buck et al., 1996) was applied to the sections of NAT/scar samples. The clinicopathological characteristics of USD patients are summarized in Supplementary Table S1, and the histological features of USD are presented in Figure 1. Participants had a median age of 45 years old (range 19–76 years old) (Supplementary Figure S1A).

**FIGURE 2:**
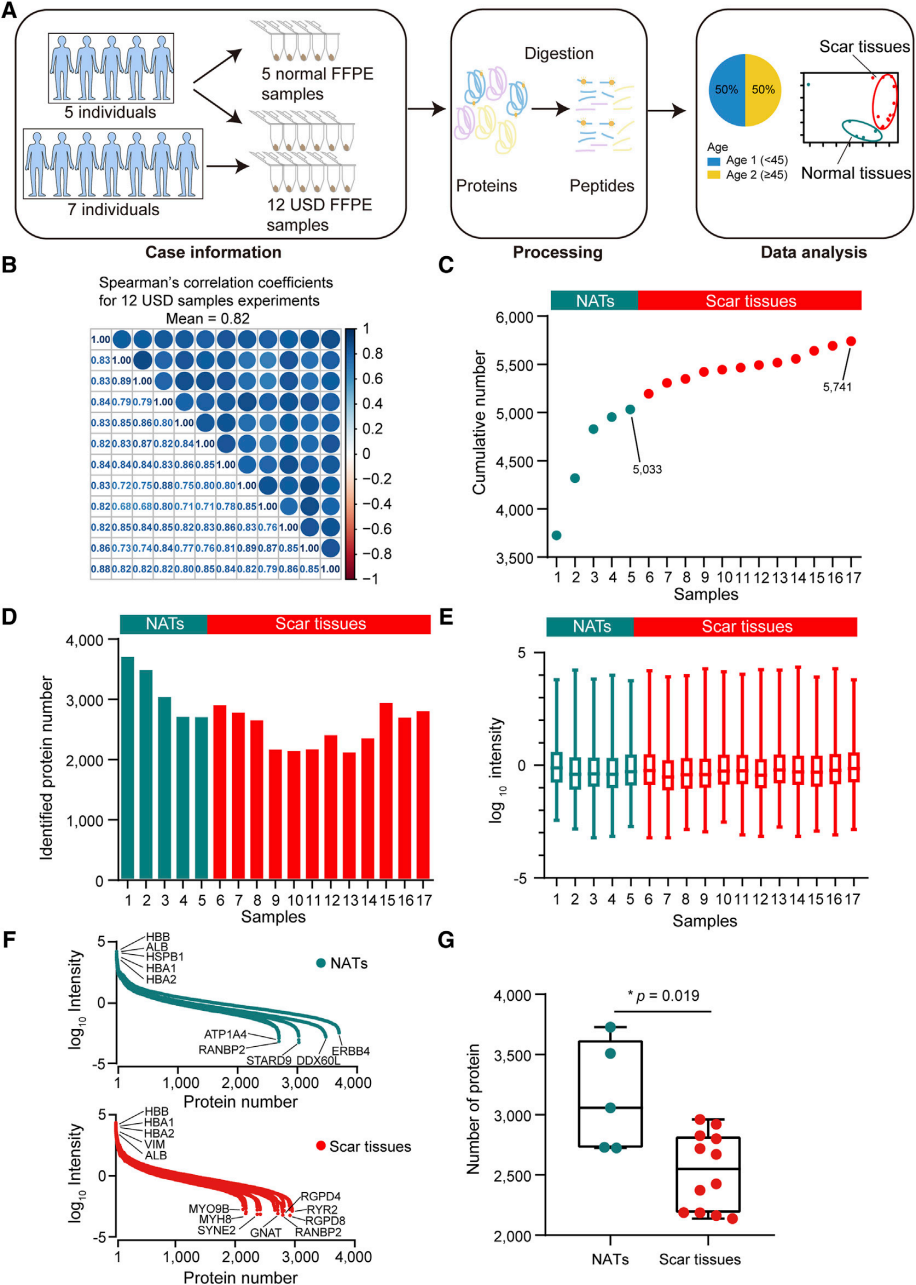
Proteomic landscape of USD. **(A)** Brief summary of case information, experiment processing, and data analysis of USD at the protein level. The 17 samples included 5 NATs and 12 scar tissues samples. **(B)** Correlation matrix of 12 USD proteomes (Spearman’s correlation coefficients). **(C)** Cumulative number of identified proteins in NATs scar tissues samples. **(D)** Bar plot indicating the number of identified proteins detected in NATs and scar tissues samples. **(E)** Boxplot illustrating the identified protein abundance (log_10_ intensity) in NATs and scar tissues samples that passed quality control. **(F)** Dynamics of protein abundance identified in NATs (top) and scar tissue (bottom) samples. Proteins were transformed to log_10_ intensity. Highest- and lowest-abundance proteins shown. **(G)** Boxplot showing the number of identified proteins in the NATs (left) and scar tissue (right) samples (Wilcoxon rank-sum test). Error bars represented min to max. ****p < 1.0 × 10^−4^, ***p < 1.0 × 10^−3^, **p < 0.01, *p < 0.05.

For proteomic data analysis, Spearman’s correlation coefficient was calculated for all QC runs using HEK293T cell samples (Supplementary Figure S1B). The average correlation coefficient of the QC samples was 0.96 (range 0.92–0.99), demonstrating consistent stability of the MS platform. To characterize the molecular mechanism of USD, we performed a comprehensive proteomic analysis of twelve scar tissues and matched five NATs using label-free global proteome profiling. A schematic overview of the experimental design is shown in Figure 2A. High technical reproducibility was evidenced by a (Spearman’s) mean correlation of 0.82 and 0.79 for the twelve scar tissues and five NATs, respectively (Figure 2B and Supplementary Figure S1C). Label-free quantification measurement of 17 samples resulted in 5,741 protein groups with a 1% FDR at the protein and peptide levels, in which 5,033 and 4,483 proteins were identified in the NATs and scar tissues, respectively (Figures 2C,D; Supplementary Figure S1D). Furthermore, the reference proteome was highly dynamic based on protein abundance spanning over eight orders of magnitude (Figure 2E). Top protein, such as HBB, HBA1, HBA2, and ALB, were extensively expressed in the NATs and scar tissue (Figure 2F). Conversely, low abundance proteins were different between the NATs and scar tissues—ATP1A4, RANBP2, STARD9, DDX60L, ERBB4, and others were detected in the NATs (Figure 2F). In the scar tissues, low abundance proteins including MYH8, GNAT, RGPD8, and RYR2 were not recorded in the NATs (Figure 2F). Compared to the scar tissues, more proteins were identified in the NATs (Wilcoxon rank-sum test, *p* = 0.019) (Figure 2G; Supplementary Figure S1E), indicating that the key events happened during scar formation.

### Scar-associated proteomic events

**FIGURE 3:**
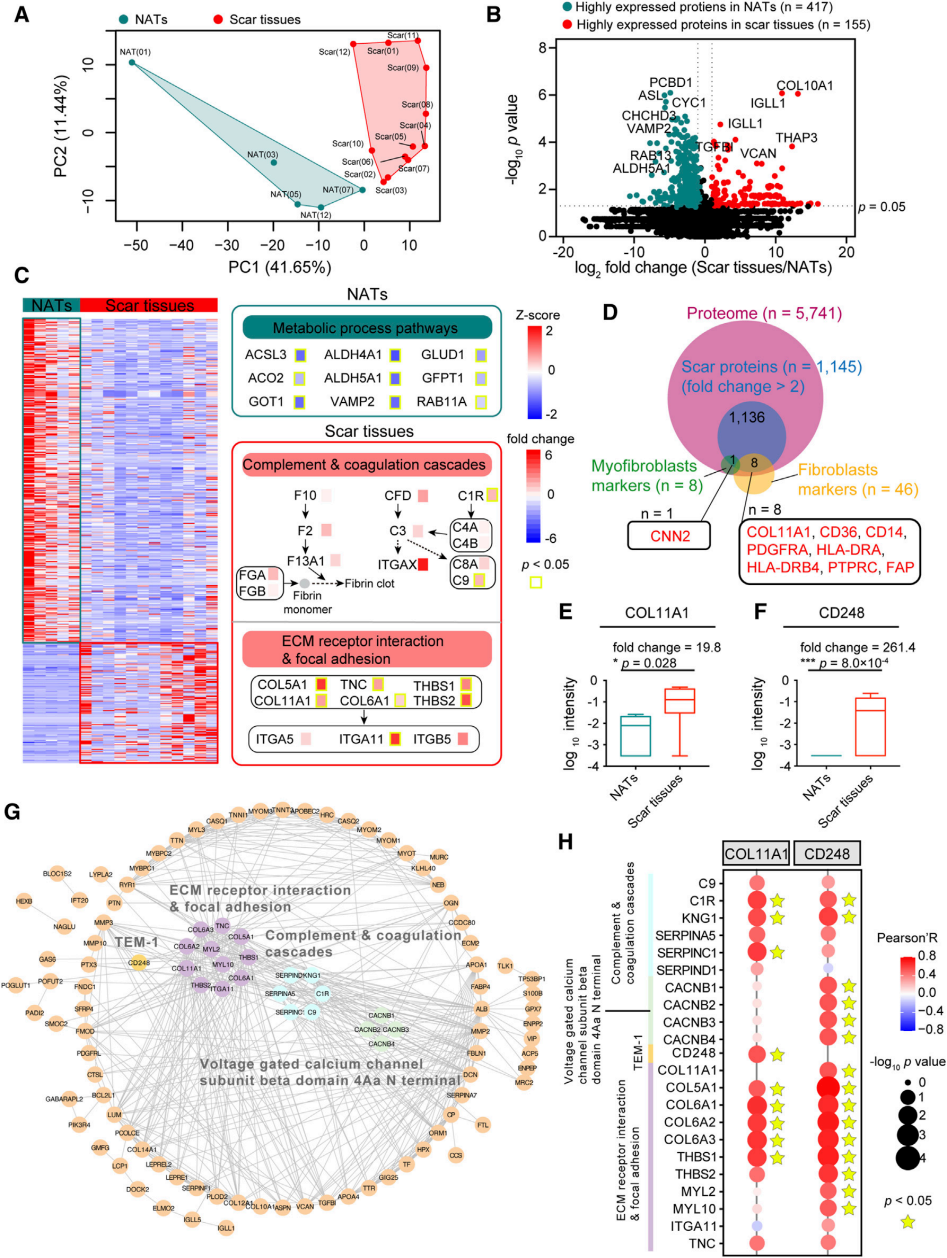
Scar-associated proteomic events. **(A)** PCA analysis of NATs (green) and scar tissues (red). **(B)** Volcano analysis depicting the differentially expressed proteins of NATs (green) and scar tissues (red). **(C)** KEGG enrichment representing the dominant pathways in the NATs. Yellow boxes indicate statistically significant differences (*p* < 0.05). **(D)** Venn diagram depicting overlaps of the scar-associated myofibroblast and fibroblasts markers. **(E)** Boxplot showing the log_10_ intensity of COL11A1 in NATs and scar tissues (Wilcoxon rank-sum test). **(F)** Boxplot showing the log_10_ intensity of CD248 in NATs and scar tissues (Wilcoxon rank-sum test). **(G)** Protein–protein interaction (PPI) analysis of the 155 differentially expressed proteins. **(H)** Correlation analysis (Pearson’s correlation test) between COL11A1/CD248 expression and the dominant pathways in the scar tissues. Error bars represent min to max. ****p < 1.0 × 10^−4^, ***p < 1.0 × 10^−3^, **p < 0.01, *p < 0.05.

To explore the difference between the NATs and scar tissues, we performed principal component analysis (PCA) at the protein level. PCA visualization clearly discriminated between the proteomes of the NATs and scar tissues (Figure 3A). To investigate specific characteristics of the NATs and scar tissues, we applied significance analysis of microarray (SAM) and identified 572 differentially expressed proteins (DEPs) between the NATs and scar tissues (Wilcoxon rank-sum test, *p* < 0.05, fold change (scar tissues/NATs) > 2 or ≤0.5), including 155 elevated (scar proteins) and 417 descend proteins (NATs proteins) (Figure 3B; Supplementary Table S2).

KEGG enriched analysis indicated that the NATs proteins were related to the metabolic process pathways (e.g., ACSL3, ALDH4A1, etc.), including alanine, aspirate, and glutamate metabolism (*p* = 2.2 × 10^−5^), fatty acid degradation (*p* = 8.7 × 10^−5^), and vasopressin-regulated water reabsorption (*p* = 1.2 × 10^−4^) (Figure 3C; Supplementary Figure S2A). Specifically, levels of PADI1 and PADI3 decreased in scar formation at the protein level (Supplementary Figure S2B). Observation of the KEGG enriched analysis indicated that the scar proteins participated in complement and coagulation cascades (*p* = 6.4 × 10^−4^) (e.g., C1R, C9, etc.), extracellular matrix (ECM)-receptor interaction (*p* = 2.4 × 10^−6^), and focal adhesion (*p* = 3.9 × 10^−5^) (e.g., COL11A1, ITGA11) (Figure 3C; Supplementary Figure S2A). Generally, extrafollicular HIC1 accelerates wound closure and generates a fibrotic scar following deep skin injury. In our study, we found that the expression of HIC1 was enhanced during scar formation (Supplementary Figure S2C). Notably, the increased expression of DPP4 (Supplementary Figure S2D) suggested the potential functions of fibroblasts and myofibroblasts in USD.

To investigate how urethral injury lead to fibrotic scars and to identify potential therapeutic targets, we integrated the highly expressed proteins (n = 1,145, fold change (scar tissues/NATs) > 2)) of the scar tissues and matched them to the fibroblast and myofibroblast makers (referenced in the CellMarker web: http://bio-bigdata.hrbmu.edu.cn/CellMarker/) (Figure 3D). Among the scar-associated myofibroblasts markers, CNN2 (fold change (scar tissues/NATs) = 2.56) was more highly expressed in the scar tissues than the NATs (Supplementary Figure S2E). Overrepresented COL11A1 (Wilcoxon rank-sum test, *p* = 0.028) was the one protein of the scar-associated fibroblast markers (Figure 3E), suggesting a potential therapeutic strategy of inhibiting COL11A1 for USD. It is notable that the proteome level of CD248 (Wilcoxon rank-sum test, *p* = 8.0 × 10^−4^) was significantly enhanced during scar formation in USD (Figure 3F).

Protein domain enrichment analysis was performed to identify the functional domains of the scar proteins. As illustrated in Supplementary Figure S2F, the voltage-gated calcium channel subunit beta domain 4Aa N terminal (4/4, FDR = 7.3 × 10^−4^) is significantly enriched in the Pfam database (Supplementary Figure S2F). Protein–protein interactions (PPIs), ubiquitous in biological systems and often dysregulated in disease, were analyzed to elucidate the DEPs in scar tissues (Figure 3G). The PPI network showed that extracellular matrices (e.g., COL11A1, COL5A1, THBS1) and complement (e.g., C9, C1R, SERPINC1) associated proteins were upregulated in scar tissues. Moreover, all four family members at the N-terminus of the voltage-gated calcium channel subunit β domain 4Aa were upregulated in scar tissues, including CACNB1, CACNB2, CACNB3, and CACNB4. CD248—also known as endosialin and TEM-1—which plays a crucial role in myofibroblast generation and accumulation, was highly expressed in scar tissues (Figure 3G; Supplementary Figure S2G). To further demonstrate this speculation, we performed a correlation analysis by integrating COL11A1 and CD248 with proteins involved in complement and coagulation cascades, the voltage-gated calcium channel subunit beta domain 4Aa N terminal, and the ECM receptor interaction and focal adhesion. As a result, we found that COL11A1 and CD248 were positively correlated with the significant makers in the complement and coagulation cascades (C1R, KNG1, and SERPINC1) and ECM receptor interaction and focal adhesion signaling (COL5A1, COL6A1, and THBS1) (Figure 3H). This analysis demonstrated that scar formation is characterized by elevated levels of complementary and extracellular matrices, with overactivation of the voltage-gated calcium channel and CD248.

### Proteomic characteristics of USD with different ages

**FIGURE 4:**
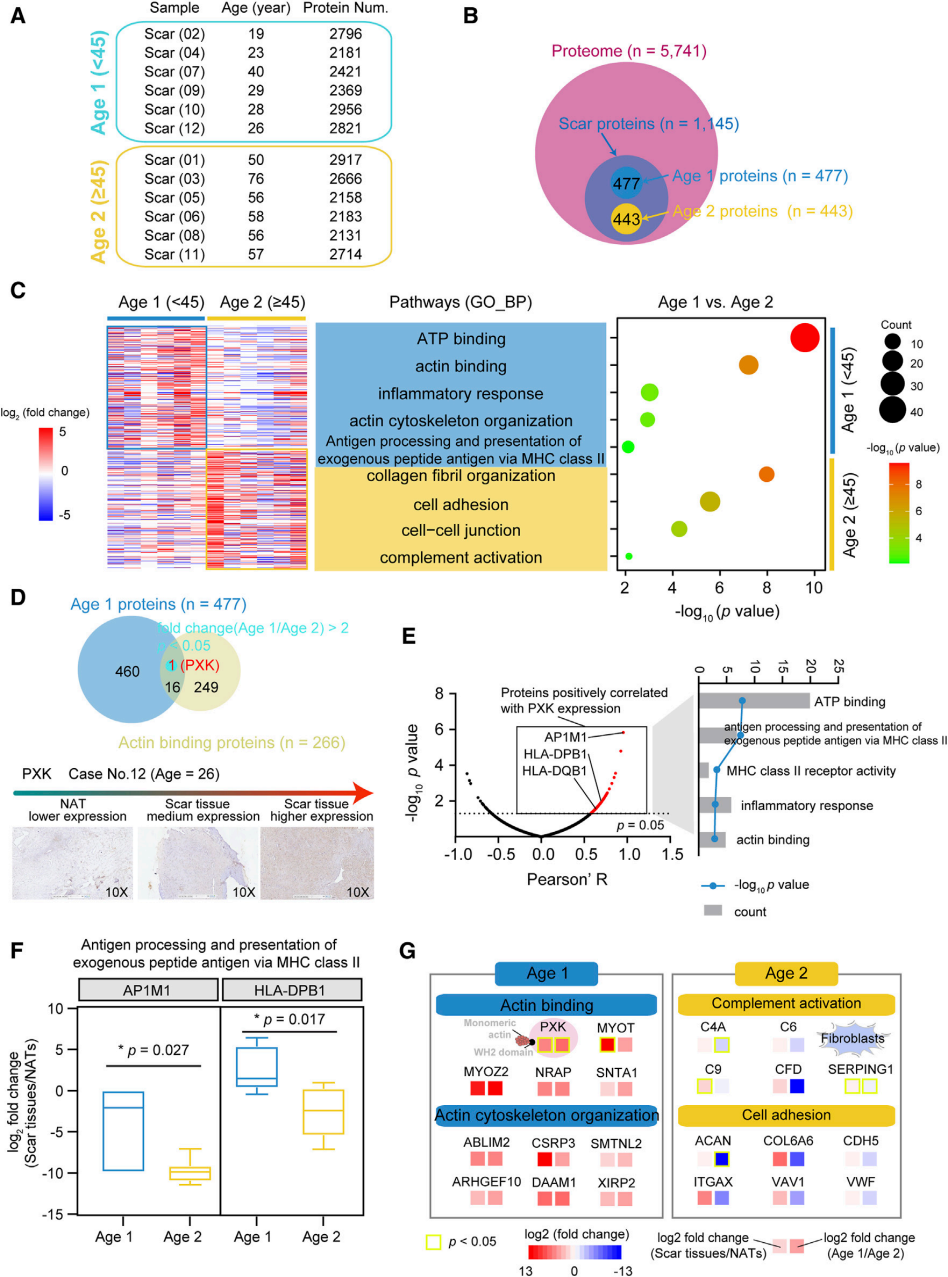
Proteomic profiles associated with ages of USD patients. **(A)** Protein number of identified protein in whole-tissue extraction in Age 1 (<45) and Age 2 (≥45) patients. **(B)** Venn diagram depicting overlapped proteins of Age 1 (fold change (Age 1/Age 2) > 2 and fold change (NATs/scar tissue) > 2) & Age 2 (fold change (Age 2/Age 1) > 2 and fold change (NATs/scar tissue) > 2) patients at the protein level. **(C)** Heatmap of supervised hierarchical clustering (left) and pathway enrichment analysis (right) showing the differences between Age 1 and 2 patients. **(D)** Venn diagram (top) of Age 1 proteins and actin binding proteins, and IHC staining (bottom) of PXK in Age 1 patients (Wilcoxon Rank-sum test). Red represents overlapped genes (*p* < 0.05). **(E)** Volcano plot depicting PXK positive associated proteins (left), and associated signaling pathways (right). **(F)** Boxplots showing log_2_ fold change (scar tissues/NATs) of AP1M1/HLA-DPB1 in Age 1 and 2 patients. **(G)** Brief summary of pathways enrichment in Age 1 and 2 patients. Red boxes indicate statistically significant differences (*p* < 0.05). Yellow boxes indicate statistically significant differences (*p* < 0.05). Error bars represent min to max. ****p < 1.0 × 10^−4^, ***p < 1.0 × 10^−3^, **p < 0.01, *p < 0.05.

The USD patients in our cohort had a median age of 45, with more proteins (median = 2,609) detected in the Age 1 patients (<45) (Figure 4A; Supplementary Figure S3A). To characterize the proteomic profiles of USD patients of different ages, we integrated the Age 1 specific proteins (n = 477, fold change (Age 1/Age 2) > 2 and fold change (scar tissue/NATs) > 2) and Age 2 specific proteins (n = 443, fold change (Age 1/Age 2) ≤ 0.5 and fold change (scar tissue/NATs) > 2) and then performed GO enrichment analysis (Figure 4B). We thus determined that the Age 1 specific proteins were involved in ATP binding (*p* = 2.5 × 10^−10^), actin binding (*p* = 6.1 × 10^−8^), inflammatory response (*p* = 9.5 × 10^−4^), actin cytoskeleton organization (*p* = 1.2 × 10^−3^), and antigen processing and the presentation of exogenous peptide antigen via MHC class II (*p* = 7.7 × 10^−3^) (Figure 4C). Collagen fibril organization (*p* = 1.1 × 10^−8^), cell adhesion (*p* = 2.6 × 10^−6^), cell–cell junction (*p* = 5.3 × 10^−5^), and complement activation (*p* = 7.2 × 10^−3^) were the dominant pathways in the Age 2 patients, evidenced by the overrepresentation of C4A (Wilcoxon Rank-sum test, *p* = 0.278 (scar tissues/NATs) and 1.4 × 10^−3^ (Age 1/Age 2)), and SERPING1 (Wilcoxon Rank-sum test, *p* = 0.041 (scar tissues/NATs) and 0.045 (Age 1/Age 2)) (Supplementary Figures S3B,C).

To further investigate the potential markers of the Age 1 patients, we incorporated the Age 1 specific proteins and the actin binding proteins (n = 266) (referenced in the AmiGO 2 web: http://amigo.geneontology.org/). Only PXK (Wilcoxon rank-sum test, *p* = 0.041 (scar tissues/NATs) and 0.028 (Age 1/Age 2)) showed significant overrepresentation in the Age 1 patients, which was also corroborated by IHC staining (Figure 4D and Supplementary Figure S3D). In our cohort, the upregulated PXK had positive impacts on the proteome-level of antigen processing and the presentation of exogenous peptide antigen via MHC class II and actin binding, as evidenced by the adaptor-related protein complex 1 Subunit Mu 1 (AP1M1) and MHC II molecules (HLA-DPB1 and HLA-DQB1) (Figure 4E). Compared to the Age 2 patients, the protein level of AP1M1 (Wilcoxon Rank-sum test, *p* = 0.027) and HLA-DPB1 (Wilcoxon Rank-sum test, *p* = 0.017) were overrepresented in Age 1 (Figure 4F). We thus revealed the diverse proteomic profiling of USD patients with different ages, disclosed the characterized pathways, and identified potential markers in the Age 1 (e.g., PXK) and Age 2 (e.g., C4A, SERPING1) USD patients (Figure 4G).

### Immune infiltration of USD

**FIGURE 5:**
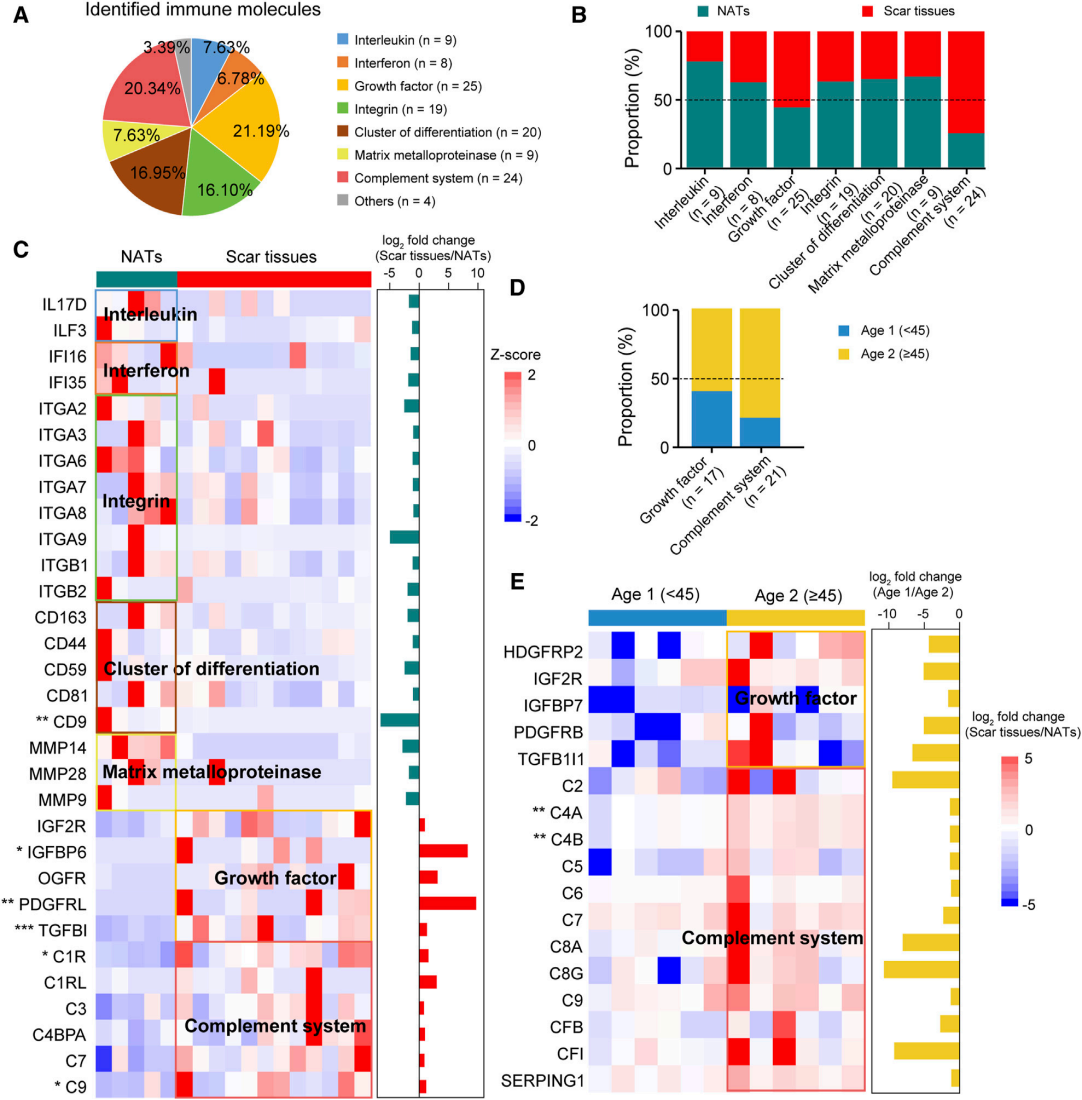
Immune characterizations of USD. **(A)** Pie chart showing proportion of immune molecules at the protein level. **(B)** Bar plot indicating proportion of immune molecules in NATs and scar tissues. **(C)** Heatmap of supervised hierarchical clustering analysis showing upregulated immune molecule in NATs and scar tissues. **(D)** Bar plot indicating proportion of cytokines in Age 1 (<45) and 2 (≥45) patients. **(E)** Heatmap representing upregulated immune molecules in Age 1 (<45) and 2 (≥45) patients.

To investigate immune infiltration in USD, we accessed the Human Protein Atlas (HPA) immune protein database (https://www.proteinatlas.org) and found that 118 immune molecules were detected in our cohort, including 25 growth factors and 24 complement system related proteins (Figure 5A Supplementary Table S3). Comparative analysis elucidated a higher representation of interleukins (e.g., IL17D, ILF3, etc.), interferons (e.g., IFI16, IFI35, etc.), integrins (e.g., ITGA2, ITGA3, ITGA6, etc.), CD molecules (e.g., CD163, CD44, CD59, etc.), and matrix metalloproteinases (MMPs—e.g., MMP14, MMP28, MMP9, etc.) observed in the NATs (Figures 5B,C). In the scar tissues, growth factor- and complement system-correlated proteins were overrepresented, including IGFBP6 (Wilcoxon rank-sum test, *p* = 0.040), PDGFRRL (Wilcoxon rank-sum test, *p* = 2.7 × 10^−3^), TGFBI (Wilcoxon rank-sum test, *p* = 1.5 × 10^−4^), C1R (Wilcoxon rank-sum test, *p* = 0.015), and C9 (Wilcoxon rank-sum test, *p* = 0.045) (Figure 5C).

Overrepresentation of growth factors and complementary system-correlated proteins were observed in the Age 2 patients (Figure 5D). Specifically, C4A (Wilcoxon rank-sum test, *p* = 1.4 × 10^−3^) and C4B (Wilcoxon rank-sum test, *p* = 1.4 × 10^−3^) showed significant impacts, indicating the medicative effect of eculizumab (anti-complementary) for the Age 2 patients (Figure 5E). These results suggested that immune protein inhibition could be a potential therapeutic strategy for USD.

**FIGURE 6:**
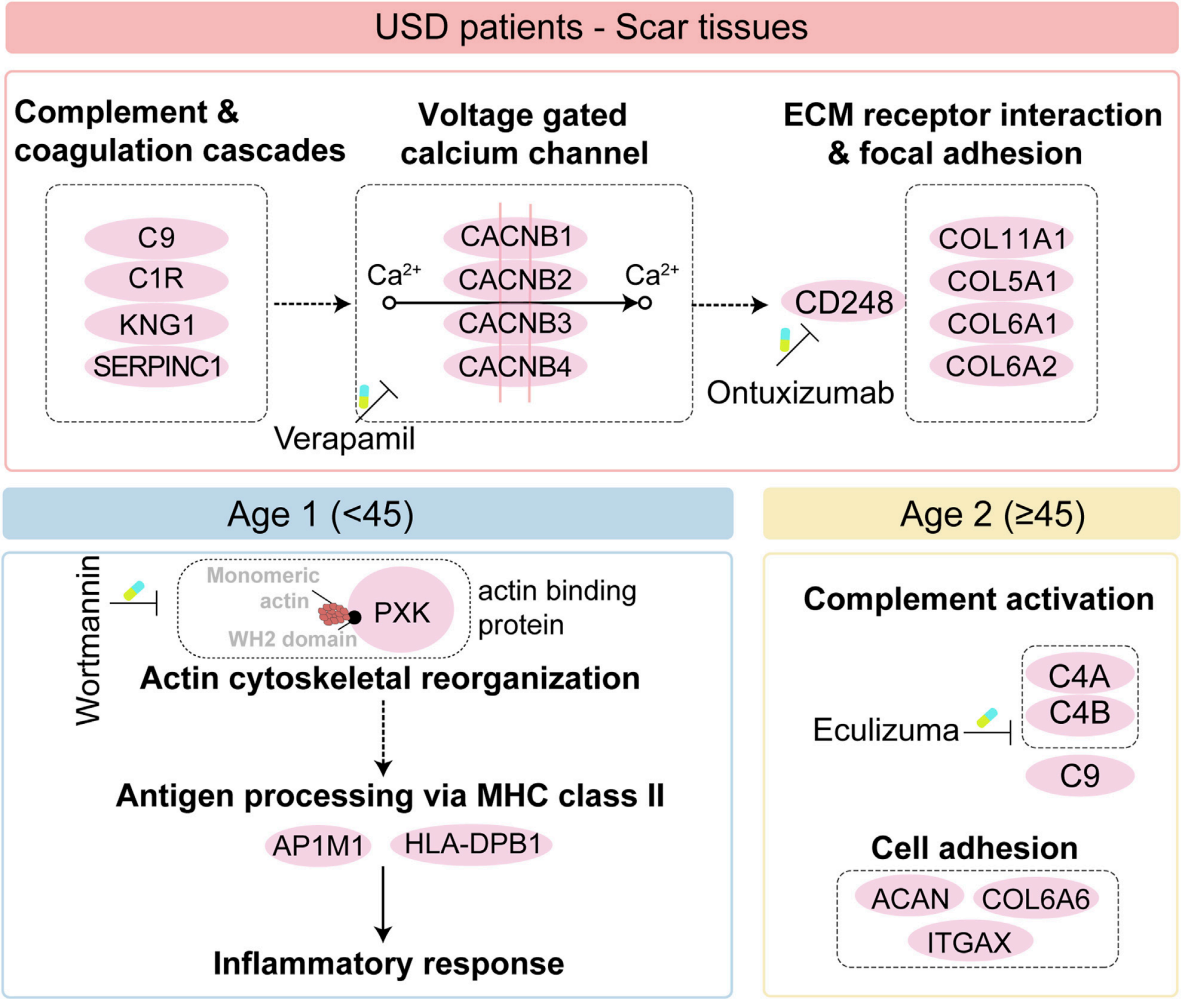
Brief summary of the disease mechanism and medicative drug targets in all USD patients (top), and the Age 1/ 2 patients (bottom).

Taken together, we illustrated the immune infiltration of USD and nominated the potential drug and targets of the scar formation for the Age 1 patients (wortmannin to anti-PXK) and Age 2 patients (eculizumab to anti-complementary). Importantly, we illustrated the pathogenesis of all USD, and the differences in different age groups also give us a broader understanding of pathogenesis (Figure 6).

## Discussion

USD is preventable among men (Santucci et al., 2007). Surgical intervention, such as urethral dilatation, endoscopic urethrotomy, and urethroplasty, aim to restore normal voiding but are often accompanied by recurrent issues and various complications (Shahrour et al., 2018). The deregulation of ECM synthesis and production may result in fibrosis and scarring (Demyanenko et al., 2015; Zhao et al., 2017). Generally, fibroblasts and myofibroblasts are responsible for depositing the collagen and elastic fibers of the ECM (Kalluri and Zeisberg, 2006). Distinct fibroblast lineages are critical in determining the dermal architecture during skin development and repair, which is often accompanied by scar formation (Driskell et al., 2013; Chen et al., 2022). There is currently no curative treatment for USD.

Mass spectrometry (MS)-based label-free quantification strategy (Ge et al., 2018; Jiang et al., 2019; Anwaier et al., 2022; Mill and Li, 2022) is widely applied for depth presenting molecular characterizations of certain diseases, promoting us to explore the scar formation of USD. Our study provided a comprehensive proteomic landscape of USD to investigate the molecular characterization of scar formation. The distinct separation between NATs and scar tissues indicated the key events occurring during scar formation. Comparative analysis of DEPs illustrated the decline of metabolic processes and the enhancement of ECM signaling and complement cascades. The chronically activated fibroblast and myofibroblast lineages deposit collagens and fibers of ECM (Kalluri and Zeisberg, 2006; Pinar et al., 2020), stimulating urethral repair to facilitate wound healing and scar formation (Rinkevich et al., 2015). The inhibition of DPP4, a component of elastic fibers in the ECM, results in diminished cutaneous scarring during wound healing (Rinkevich et al., 2015). Scar-associated proteins, such as DPP4 and HIC1, were overrepresented in the scar tissues. Calponin (CNN) acts as an actin binding protein involved in regulating the structure and function of the actin cytoskeleton by inhibiting actin-activated myosin ATPase and motor activities (Abe et al., 1990; Hsieh et al., 2022). The integrated analysis of scar tissues and fibroblast/myofibroblast markers elucidated the potential inhibiting effects of CNN2 and COL11A1 in the therapeutic strategy for USD.

Our study illustrated for the first time the pathogenesis of USD across different age groups, providing a broader understanding of this process. The classical complement pathway requires both calcium and magnesium ions (Snyderman and Pike, 1975; El Sissy et al., 2019), enhancing calcium uptake via voltage-gated calcium and promoting collagen production during fibrosis (Shearer et al., 1976; Mukherjee et al., 2015). The calcium channel blocker verapamil effectively reduces fibrosis and scar formation (Berman et al., 2017; Khattab et al., 2020). CD248, also known as endosialin and TEM-1—a type I transmembrane molecule expressed in stromal cells—binds to ECM components (Bartis et al., 2016; Wilhelm et al., 2016) and plays a key role in myofibroblast generation and accumulation. Ontuxizumab, a CD248 inhibition, binds to human endosialin on cells expressing the antigen (Grothey et al., 2018) and reduces fibrotic deposits in urethral scarring. We thus speculated that CD248, increasingly expressed in the scar tissue of USD and associated with the enhanced voltage-gated calcium channel, is a potential target in the clinical strategy for USD (Figure 6). The distinct proteomic profiles of USD patients with different ages prompted us to investigate the proteomic features of Age 1 (<45) and Age 2 (≥45). Complementary activation and cell adhesion correlated proteins were highly expressed in Age 2 patients. Notably, actin-binding proteins played a crucial role in the pathophysiology of scar formation in Age 1 patients, which might suggest an important function for PXK (PX domain containing serine/threonine kinase-like). PXK expressed by fibroblast-like cells (Thomsen et al., 2017) acts as a functional sorting nexin involved in actin cytoskeletal reorganization by delivering actin monomers to the sites of polymerization or sequestering them (Takeuchi et al., 2010). The reorganization of the actin cytoskeleton is pivotal for activating innate immunity (Li et al., 2017; Acharya et al., 2022) and facilitating the MHC class II antigen presentation pathway (Barois et al., 1998). We presented specific therapeutic targets and drugs, such as wortmannin for inhibiting PXK in Age 1 patients (Lippman, 1992) and eculizumab for targeting complements in Age 2 patients (Ricklin et al., 2018), and provided a reference database for personalized USD medication (Figure 6).

However, limits are still represented in our study. The small sample size and trace amounts of samples are the main limitations, allowing some variability in the reported data. To assess data variability, we evaluated the expression of several standard housekeeping genes (tubblin, GAPDH, and actin). The constant expression of housekeeping genes in NATs and scar tissues indicates that the variance within our samples is within an acceptable range. Owing to the lack of the multi-omics data of USD in previous studies, we plan to collect more samples of USD and perform comprehensive characterizations at the multi-omics level in the future. As for the specificity of these biomarkers to USD, our proteomic analysis indicates a strong association with fibrotic pathways specific to urethral scar formation. However, it is important to note that while these biomarkers show promise, their specificity to USD *versus* other fibrotic conditions remains to be fully validated. We are currently conducting follow-up studies involving larger cohorts and comparative analyses with other fibrotic diseases to address this question. These studies specifically aim to delineate the expression profiles of these biomarkers in USD, thereby enhancing their diagnostic utility and specificity. Additionally, we are developing a clinical diagnostic kit that incorporates identified biomarkers. This kit aims to facilitate early detection and improved management of USD by providing a non-invasive, reliable, and quick diagnostic tool. We believe that these diagnostic markers, once validated in a larger cohort and included in a clinical diagnostic kit, could significantly improve the diagnostic accuracy and management of USD, ultimately benefiting patients through personalized therapeutic strategies.

## Conclusion

Our study presented a comprehensive proteomic landscape of USD. We disclosed the functions of ECM signaling and complement cascade in scar formation and highlighted the impacts of COL11A1 and CD248 in USD patients. In addition, we uncovered the diverse molecular mechanism of scar formation in USD across different ages and deconvoluted the immune infiltration of USD. We illuminated drug-targetable PXK in Age 1 patients and the complementary system (C4A, C4B) in Age 2 patients, demonstrating the value of this proteomics mapping strategy in suppressing scar growth. This study provides new insights into the mechanisms of fibrotic scar formation and enables advances to promote the diagnostics and therapeutics to manage USD.

## Data Availability

The datasets presented in this study can be found in online repositories. The names of the repository/repositories and accession number(s) can be found in the article/Supplementary Material.
