# Supplementary figures and images for: Comprehensive proteomic characterization of urethral stricture disease in the Chinese population
Source: Front Mol Biosci. 2024 Jul 26;11:1401970. doi: 10.3389/fmolb.2024.1401970 (PMC11310122; doi:10.3389/fmolb.2024.1401970)

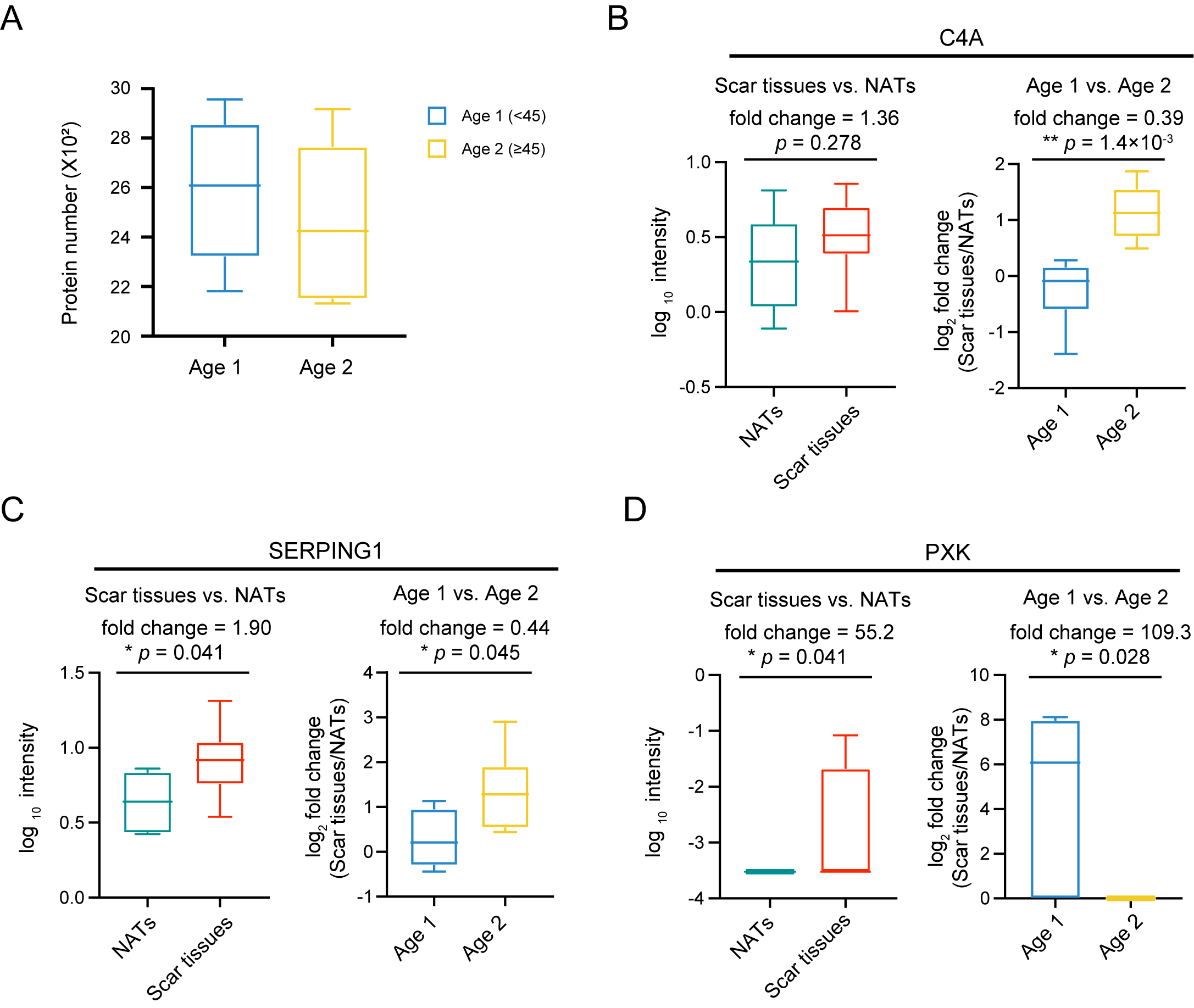

Supplement: Supplementary file 3 [file Image3.TIF]

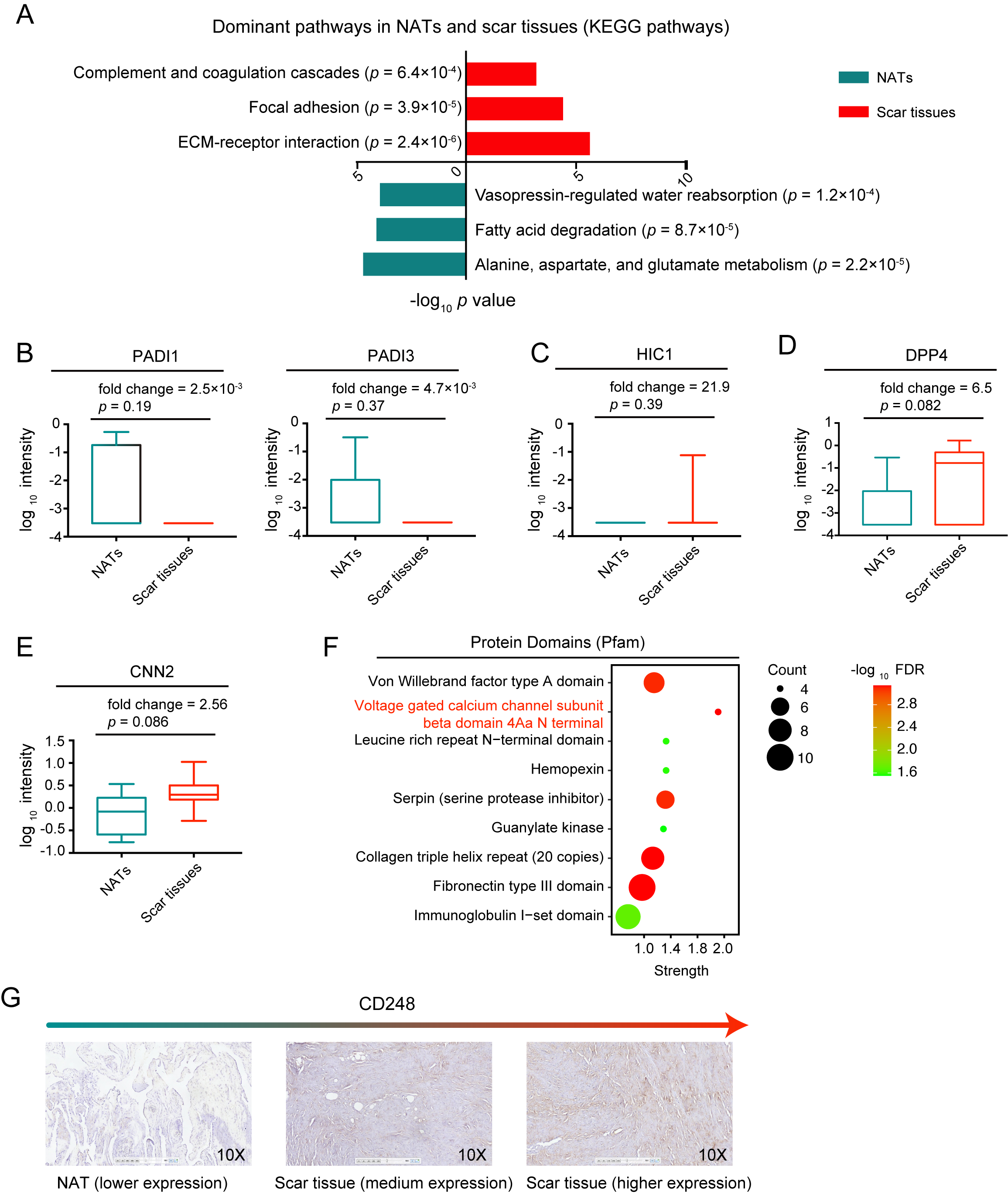

Supplement: Supplementary file 4 [file Image2.TIF]

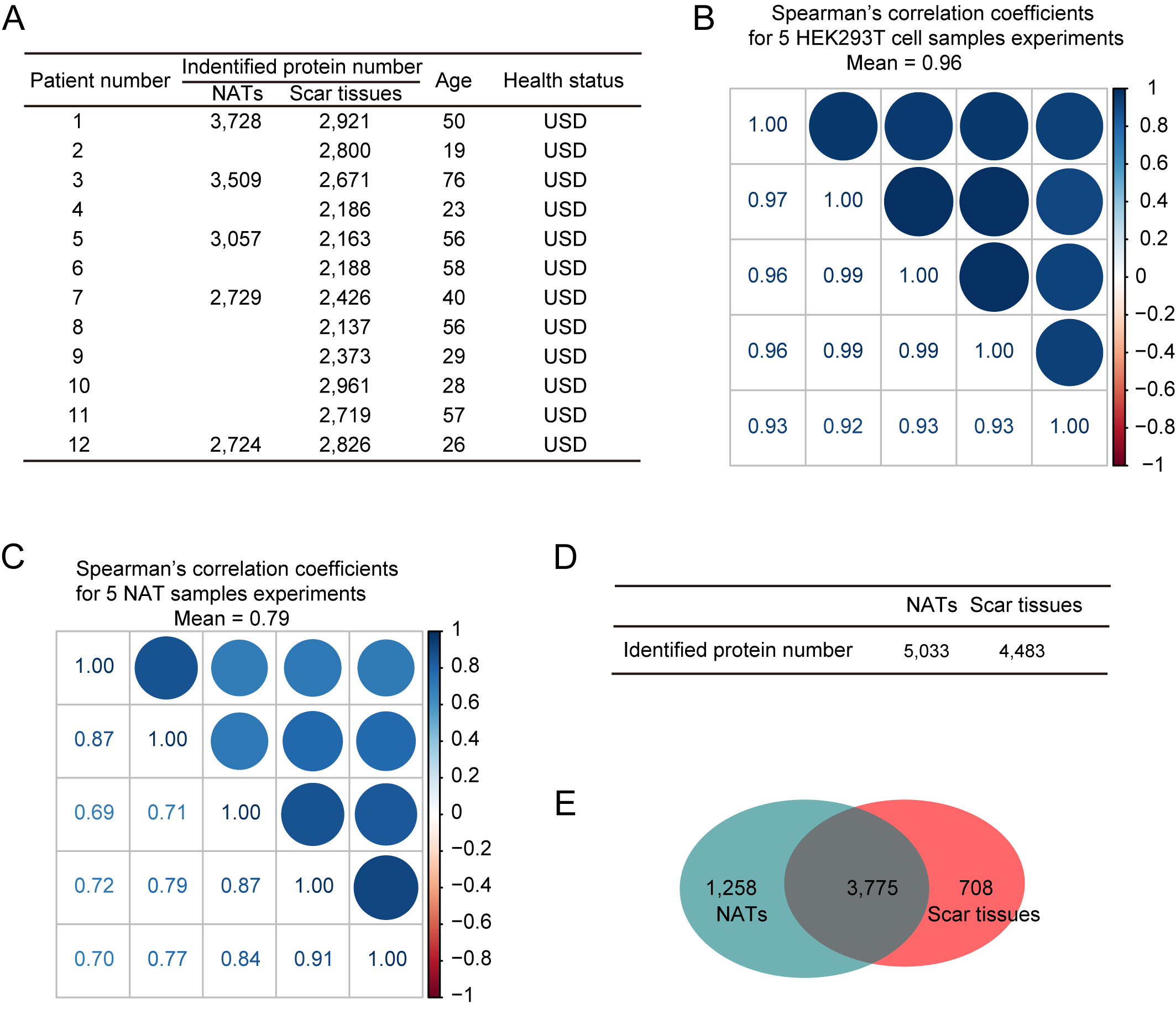

Supplement: Supplementary file 5 [file Image1.TIF]
